# Antibiotic Resistance and Probiotics: Knowledge Gaps, Market Overview and Preliminary Screening

**DOI:** 10.3390/antibiotics12081281

**Published:** 2023-08-03

**Authors:** Gordana Zavišić, Milka Popović, Svetlana Stojkov, Deana Medić, Vera Gusman, Nataša Jovanović Lješković, Aleksandra Jovanović Galović

**Affiliations:** 1 Faculty of Pharmacy Novi Sad, University Business Academy, Trg Mladenaca 5, 21000 Novi Sad, Serbia; gordana.zavisic@faculty-pharmacy.com (G.Z.); svetlana.stojkov@faculty-pharmacy.com (S.S.); natasa.ljeskovic@faculty-pharmacy.com (N.J.L.); 2 Department of Hygiene, Faculty of Medicine, University of Novi Sad, Hajduk Veljkova 3, 21000 Novi Sad, Serbia; milka.popovic@mf.uns.ac.rs; 3 Center for Hygiene and Human Ecology, Institute of Public Health of Vojvodina, Futoška 121, 21000 Novi Sad, Serbia; vera.gusman@mf.uns.ac.rs; 4 College of Vocational Studies for the Education of Preschool Teachers and Sports Trainers in Subotica, Banijska 67, 24000 Subotica, Serbia; 5 Department of Microbiology, Faculty of Medicine, University of Novi Sad, Hajduk Veljkova 3, 21000 Novi Sad, Serbia; deana.medic@mf.uns.ac.rs; 6 Center for Microbiology, Institute of Public Health of Vojvodina, Futoška 121, 21000 Novi Sad, Serbia

**Keywords:** probiotic, *pharmacobiotic*, antibiotic resistance, market analysis, antibiotic resistance screening, dietary supplements, *Lactobacillus*

## Abstract

Probiotics are among those products, the use of which is increasing, and they are available primarily as food/dietary supplements, as well as in the form of medicines. This study aims to assess the attitudes and practices of health professionals and students of health sciences, give a short overview of the probiotics currently on the market, and conduct a screening of five food supplements and one drug with respect to antibiotic resistance. Nearly half of the respondents in our survey state that probiotics have no side effects, while only 6.3% believe that the use of probiotics can lead to antibiotic resistance. In addition, more than 40% of the participants throw unused probiotics into municipal waste. The market analysis results indicate that probiotic products on the Serbian market have highly variable CFU counts, while the declared health claims cover numerous beneficial health effects, and they are sometimes even registered as medicines. Lactobacilli are frequently present in probiotic supplements, and are sold in pharmacies and online. The experimental results showed that antibiotic resistance is present in different types of lactobacilli in probiotic products. The risk of using probiotics, regardless of their beneficial health effects, should be taken into account in the future. An update to the regulations governing probiotics, including a stipulation for antimicrobial resistance (AMR) testing, should be established, and guidelines for their proper use and disposal put into place.

## 1. Introduction

The market for dietary products and supplements has been growing for several decades. Probiotics are among those products, the use of which is increasing year by year, and they are available in a large number of forms—primarily as food/dietary supplements (tablets, capsules, powder, ampoules), but also as medicines (*pharmacobiotics*), as well as being present in cosmetic products (certain forms). According to the International Probiotic Association (IPA), the global market for probiotic supplements was valued at almost USD 7 billion in 2021, with a forecast growth of 3.75% on average until 2026 [1]. The IPA states that the global probiotics market size grew from USD 66.9 billion in 2022 to USD 73.14 billion in 2023, with a compound annual growth rate (CAGR) of 9.3%. According to the same report, increasing consumer preference for healthy foods and nutritional supplements is expected to fuel the growth of the probiotics market in the future. Taking care of one’s own health has developed into an important component of the lifestyle of modern consumers. Nevertheless, the influence of marketing cannot be ruled out, with the pharmaceutical market increasingly gaining a commercial aspect at the expense of the efficiency and safety of the products it promotes [2].

The definition of probiotics, dating to 2001, and given by the World Health Organization (WHO) and the U.S. Food and Drug Administration (FDA), states that “probiotics are live microorganisms that, applied in adequate amounts, provide a health benefit to the host” [3]. It was amended in 2014 with an additional requirement saying that probiotics must have “a defined content, an appropriate number of viable cells at the end of the product’s shelf life, and adequate evidence of health benefits, and must be safe for their intended use” [4].

Binda et al. [5] state the basic requirements for probiotic products as follows: strains must be sufficiently, clearly and unambiguously characterized; safe for intended use (supported by a valid clinical trial); and should contain viable microorganisms within their shelf life. Another important criterion for microbiological quality, in addition to the identification of declared probiotic strains, is the determination of the number of viable cells (probiotic bacteria and/or fungi). There is also the requirement for the absence of specific pathogenic/opportunistic pathogenic bacteria and fungi, as well as the limitation of the total number of contaminants, such as aerobic bacteria, yeasts, and molds [6]. The absence of antibiotic resistance genes (ARG) is an important aspect of product safety. Still, there are numerous reports on the low quality of probiotics on the market [7], primarily due to a significantly smaller number of viable microorganisms, sometimes not clearly identified, or the absence of declared ones. The presence of probiotic strains carrying antimicrobial resistance (AMR) genes has also been reported [8,9]. In a study by Selvin et al. [8], the antibiotic susceptibility of samples from commercially available probiotic food supplements containing probiotics was analyzed, with strains of *Streptococcus faecalis* and *Bacillus mesentericus* showing resistance to penicillin G, while *Lactobacillus acidophilus* was resistant to ampicillin, and all isolates were resistant to ceftazidime. Consumption of probiotic products can lead to the transfer of ARGs to the human gut bacteria. Furthermore, many AMR genes (or ARGs) are associated with mobile genetic elements (MGEs) and phages. Horizontal transfer of ARGs to other bacteria may result in a reduction in the effectiveness of antibiotic treatment [9].

There is a large body of literature data documenting the plethora of health benefits of probiotics, including immunomodulatory, metabolic, anti-infective, antioxidant, anti-mutagenic, and anti-cancer activity, as well as normalization of the disturbed gastrointestinal tract–microbiota, especially after the use of antibiotics. It is important to note that health benefits are strain specific. However, often, only generalized, vague and non-specific effects of probiotics are emphasized, primarily for marketing purposes.

Based on their intended use and effect, probiotic products are regulated by the U.S. Food and Drug Administration (FDA) as a dietary supplement, food ingredient, or drug. If a probiotic is intended to cure, alleviate, treat or prevent human disease, it is classified as a drug, and must meet more stringent requirements. Although the requirements vary from country to country, the minimum amount of evidence required for a claim—obtained through appropriate trials—has to meet drug regulatory standards [10].

A probiotic, as a food or supplement without a health claim, must meet the criteria of being a member of a safe species in the EU [11], and the U.S. FDA’s Generally Recognized As Safe (GRAS) [12], as well as being supported by sufficient evidence of a general beneficial effect on humans or a safe microorganism. On the other hand, a probiotic in a food or a supplement with a specific health claim, the following criteria must be met: the probiotic must belong to a defined probiotic strain(s), and there must be proof of delivery of viable strain(s) in an effective dose at the end of the product’s shelf life [13].

Due to European health claim regulations (Regulation (EC) N°1924/2006 [14]), the use of the word ‘probiotic’ for advertising and on food supplement packaging is not allowed in most EU countries, categorizing it as an unauthorized health claim per se, in the absence of relevant scientific evidence. Serbian law on nutrition and health claims (Rulebook, 2018 [15]) also regards the term ‘probiotic’ on dietary supplements to be an unauthorized or prohibited health claim, unless used in association with an authorized health claim for a specific microorganism or other food ingredient (minerals, vitamins or herbal ingredients). At the same time, regulations on some other food categories regard the term ‘probiotic’ to be a mandatory category designation (i.e., yogurt with probiotics). Probiotics registered as medicines can also be found on the market, in which case the regulations regarding the advertising of medicines are applicable. This situation creates regulatory confusion, considering the fact that probiotics could be presented as medicines, dietary supplements, food for special medical purposes, functional foods, and food ingredients, with different regulatory frameworks regarding category designation, advertising and labeling.

With the exemption of several probiotics registered as medicines, most of the probiotics on the Serbian market are registered as food supplements. In 2022, there were a total of 147 probiotics and 38 registration holders (i.e., manufacturers or importers/distributors) on the market in the Republic of Serbia, with a total turnover of 8.1 million packages (sales channel: wholesale drugstore–pharmacy). In 2022, with respect to total turnover, 10 registration holders accounted for a participation of 91%, of which two are domestic producers, with a participation of 54%. According to the data of IQVIA [16], the number of products and the number of registration holders remained at approximately at the same level for the three years until 2022; however, a marked rise in the turnover of packages was recorded (from 5 million in 2020 to more than 8 million in 2022). This could suggest an increasing consumption of probiotic supplements in Serbia.

Most commercial probiotics contain lactobacilli (mainly from the genus *Lactobacillus*) and bifidobacteria. The genus *Lactobacillus* has been reclassified into 25 genera [17].

Our study aims to give a short overview of the probiotics currently on the market, assess the attitudes and practices of health professionals and students of health sciences, and conduct a screening of five food supplements and one drug with respect to antibiotic sensitivity/resistance. Considerations regarding the possible influence of the widespread use of probiotics in the population and their influence on the spread of antimicrobial resistance are given.

## 2. Results

### 2.1. Survey Results

A total of 541 respondents participated in this research, of whom 88.5% were female and 11.5% were male, divided into age groups of 18–25 years (33.3%), 26–50 years (60.3%) and over 50 years (6.4%). About a third of the respondents declared themselves to be a master of pharmacy (30.1%), 22% to be students of pharmacy, 20.9% to be pharmaceutical technicians, while the remaining respondents were masters of pharmacy with postgraduate education, students of health care, medical biochemistry and others. Almost half of the respondents lived in Vojvodina (46.6%), about a quarter in Belgrade (25.9%), and about 15% in Central Serbia (for detailed information on all answers, please see the Supplementary Materials, Figures S1–S7).

The majority of the respondents (82.1%) knew the exact definition of probiotics, while the answers about the most common indications for the use of probiotics and representatives of probiotic bacteria varied from 0.2% to 98.7%. More than 70% of respondents believed that probiotics should be used for seven or more days in order to achieve the optimal effect. About half (47.7%) of the respondents believed that they were partially aware of possible risks and side effects when using probiotics for certain health conditions/diseases, while the numbers of respondents who considered themselves to be fully aware and not aware were equal, ranging around 25%. As possible side effects of probiotics, respondents most often mentioned interaction with antibiotics (35.1%) and digestive disorders (23.5%), while the development of resistance to antibiotics was mentioned by only 6.3% of respondents. Almost half (49%) of respondents believed that probiotics had no side effects. The most common names of commercial probiotics known to respondents (they had used them or given recommendations for their use) were *Probiotik* (461 respondents), *Linex* (459 respondents), *Flobian* (439 respondents), *Biogaia* (332 respondents), *Biocult Candea* (296 respondents), *Liobif* (288 respondents), out of the total number of respondents (541).

Out of 541 respondents, 356 respondents (65.8%) cited formal education as a source of information, making it the most common source, 317 (58.6%) cited health professionals, and 314 (58%) respondents cited continuing medical education. Books and professional magazines were followed by 199 respondents (36.8%), websites by 160 (29.6%), while radio and television, social networks and YouTube channels were mentioned by the smallest numbers of respondents (up to 10%). The majority of respondents (93.7%) believed that their knowledge about probiotics should be supplemented and updated. When asked whether they had used probiotics in the last year, 62.6% of the respondents answered that they had used them more than once, 21.3% of the respondents had used them once; and 16.1% of respondents declared that they had not used a probiotic in the last year.

More than half of the respondents used probiotics “on their own” (51.4%) and for stomach problems (diarrhea, constipation, bloating, flatulence, etc.) (80.4%), for preventive purposes during treatment with antibiotics (78.1%), for allergic conditions (6.9%), and for stress (5.6%). At home, respondents stored the remaining quantity of probiotics until the next use (28.5%) or until the expiration date (58%). More than 90% of the participants stored probiotics in a dry, dark and cool place and checked the expiration dates before use, and if the expiration date had passed, 54.3% of the respondents would take them to the pharmacy, while 42.1% threw them in the municipal waste. The majority of respondents (88%) stated that they had given recommendations for the use of probiotics, of which 55.8% had recommended them to patients, while 32.2% had recommended them to friends and family members. As reasons for recommending taking probiotics, respondents cited antibiotic therapy (95%), diarrhea and other stomach problems (92.4%), prevention of travel diarrhea (65.5%), strengthening the immune system (59.9%), obesity (9.7%) and mood disorders (8.1%).

### 2.2. Market Analysis Results

Of the 113 probiotics registered in the Republic of Serbia analyzed, more than half (53%) were marketed as a combination of probiotics with other, different, active ingredients, such as zinc, prebiotics (inulin, FOS, GOS), vitamin D, vitamin C, different herbal ingredient, and enzymes.

**Figure 1:**
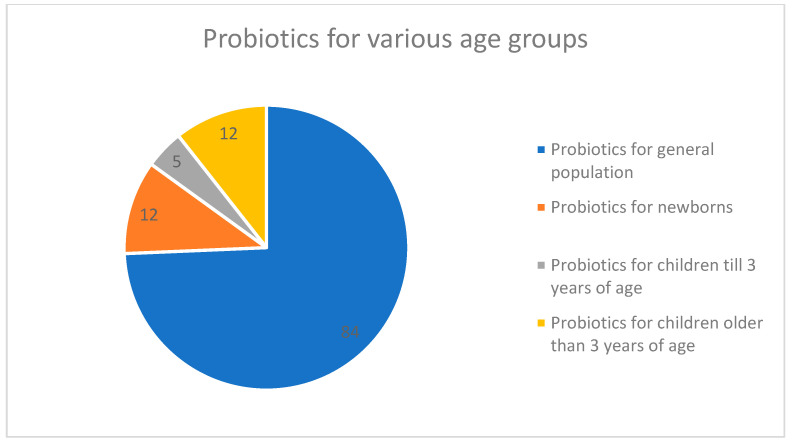
The age distribution of intended use for probiotic supplements on the market in Serbia (as labeled on the package).

Out of a total of 113 products, 84 (74.34%) were intended for the general population and 29 (25.66%) were for children, with 12 being labeled as products intended for newborns, 5 as for children <3 years of age, and 12 as for children >3 years of age (Figure 1).

It is interesting to note that the price range of the analyzed products was very wide (USD 2.4–70), with the priciest being 29-fold more costly than the cheapest. Probiotics intended for the pediatric population were generally priced higher.

**Figure 2:**
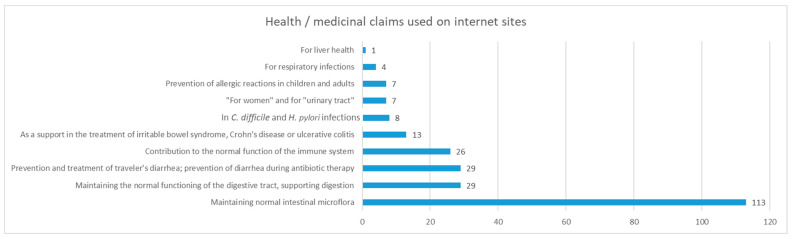
Health/medicinal claims on probiotic supplements sold online, with marketing purposes.

In our sample, the most common indications were: maintenance of normal intestinal microbiota (all analyzed supplements), prevention of diarrhea (29), digestion support (29), and contribution to immune system function (26) (Figure 2). It is important to bear in mind that the internet marketing of probiotics and other food supplements does not fall under strict governmental supervision, so medicinal claims are often used online, as opposed to for sales in pharmacies, where making such claims on the packages is prohibited.

**Figure 3:**
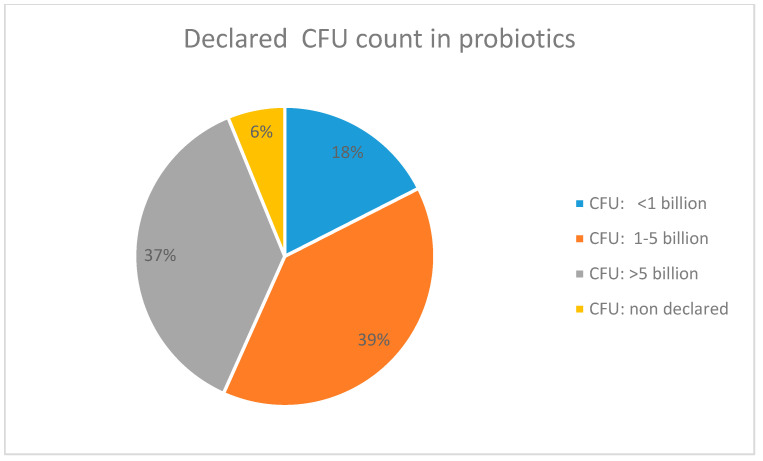
The CFU count declared on the package of probiotics sold online in Serbia.

The number of viable microorganisms, expressed as Colony-Forming Units (CFU), is one of the parameters that should be declared for all types of probiotic supplement. In our market analysis, CFU count ranged from fewer than a billion up to 100 billion or more (Figure 3). The highest percentage of the products in our study had 1–5 billion CFU, while a notable 6% did not declare the presence of CFU at all.

**Figure 4:**
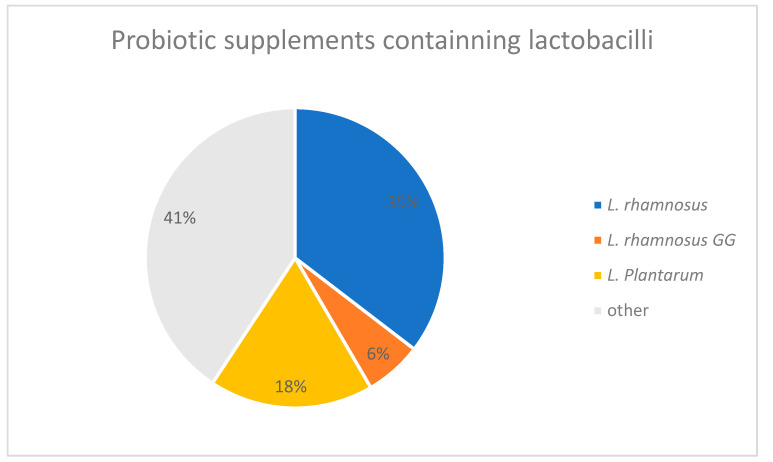
The percentage of lactobacilli-containing probiotics in relation to all other analyzed probiotic supplements on the market.

Through composition analysis of the 113 probiotic supplements included in our market analysis, 40 contained *L. rhamnosus* (of which 7 contained *L. rhamnosus* GG strain), and 20 products declared the inclusion of *L. plantarum* on the label. Together, these products account for nearly 60% of the products included in the study (Figure 4).

### 2.3. Antibiotic Sensitivity Screening Results

In our experimental work, the conventional methodology of bacterial isolation from commercial products, and the phenotypic identification of isolates and antibiotic sensitivity/resistance screening were applied.

Of the six most commonly used commercial probiotics on the Serbian market selected, five of which were dietary supplements (labeled as A, B, C, D and F) and one of which was a drug–pharmabiotic (labeled as E), after revitalization and isolation of the bacteria, a total of six isolates were morphologically and biochemically characterized at the biochemical level using MALDI-TOF, and were identified as the species *Limosilactobacillus reuteri*, *Lactobacillus paracasei*, *Lactobacillus rhamnosus*, *Lactobacillus rhamnosus*, *Enterococcus faecium* and *Lactobacillus plantarum*.

Using the disk diffusion method, each of the isolates was tested for sensitivity to 30 standard antibiotics. The results are presented in Table 1.

All isolates (lactobacilli and enterococci) were resistant to penicillin, cefoxitin, oxacillin, and aztreonam, and all lactobacilli from dietary supplements A, B, C, D, and F were also resistant to vancomycin.

It is interesting to note that pharmacobiotic (designated E) was resistant to a different set of antibiotics included in the testing, while all lactobacilli shared a similar resistance “pattern”.

## 3. Discussion

### 3.1. Knowledge, Attitudes and Practice

Although the effects of probiotics on health were recognized at the beginning of the twentieth century, new knowledge and evidence about the effects of these products on health is still being discovered. Probiotics are associated with a large number of potentially beneficial effects, and by conducting scientific research, knowledge about their spectrum of action is continuously being expanded.

Pharmacists, as the last link in the supply chain of probiotics and the most accessible health professionals for providing advice on their method of application and storage, should without a doubt have the appropriate competencies to ensure the safe, effective and rational use of these products.

Knowledge regarding the definition of probiotics as living microorganisms that, applied in adequate quantities, show beneficial effects on the host’s health [4] was shown by about 80% of the respondents in our study, which is similar to the results of the research by Fijan et al. [18], where more than 80% of pharmacists, doctors and dentists knew the correct definition of probiotics, while a smaller number of correct answers were given by nurses and psychologists.

The subjects of our study recognized *Lactobacillus acidophilus* (88.9%), *Bifidobacterium bifidum* (74.1%) and *Saccharomyces boulardii* (74.1%), *Bacillus subtilis* (24.2%) as probiotic bacteria, while fewer than 10% included *Enterococcus faecium* and *Escherichia coli* among the probiotic bacteria; 5.7% of respondents included *Mycobacterium avium* as a probiotic bacterium, which is a similar to that of an international study among health workers (4%) [18], while 2.5% of students in an Indonesian study gave the same (wrong) answer [19].

The majority of our respondents knew that optimal effects require a longer use of probiotics (71.5%); most respondents knew the required duration of probiotic therapy (70.6%), but only one-quarter believed that they were fully aware of the possible risks of side effects of probiotics in certain diseases and conditions. However, almost half stated that probiotics had no side effects, and only 6.3% believed that the use of probiotics could lead to resistance to antibiotics.

When asked how they acquired knowledge about probiotics, the respondents cited formal education (65.8%), continuing medical education (58%) and health professionals (58.6%) as the most common sources of information, followed by books and professional magazines (36.8%) and websites (29.6%), radio and television (10%), social networks (8.5%) and YouTube channels (2.6%). Other similar studies have shown that respondents generally choose reliable sources of information: academic education (71.3%) was the most common among respondents in the Indonesian study [19], while the majority (62%) of respondents in Jordan named scientific sources of information [20], and more than half of respondents in an international study cited books and journals as their primary sources of information [18].

Although the level and specificity of the respondents’ knowledge about probiotics varied, it is important to note that the majority of respondents in our study (97.3%) believed that it was necessary to supplement and update their knowledge about probiotics. A similar attitude has been shown to occur among respondents in other studies on this topic [18,19], in which 88.5% and 57.5%, respectively, of all respondents wanted to learn more about probiotics. However, the need for professional development is associated with the degree of responsibility of health professionals, but also with their abilities in terms of self-reflection and self-assessment of knowledge [21].

Knowledge about the beneficial effect of probiotics can be linked to behavior in practice: 83.9% of respondents had used probiotics in the last year, half of them had applied probiotics on the recommendation of a doctor/pharmacist, while about 80% of the research participants cited preventive purposes during treatment with antibiotics and for stomach problems (diarrhea, constipation, flatulence, etc.) as their reason. Additionally, almost all respondents (98.8%) in the study by Rahmah et al. [19] used probiotic preparations, mostly for strengthening the immune system (56.3%), prevention of GIT complaints (36.8%), and treatment of GIT complaints, while only 6.9% stated that it was for the prevention of side effects of antibiotics. The respondents in that study believed that probiotics would have a beneficial effect when taken during antibiotic therapy (90.2%), during diarrhea (83.5%), with constipation (70.6%), and before traveling abroad (63%), which somewhat coincides with our research results. About 60% of respondents stated that probiotics were effective in the treatment of allergies, while about half believed that probiotics were effective for treating depression, mood disorders, pollen allergies, cancer and liver disease.

The respondents in our study showed appropriate practices regarding expiration dates and storage at home, thus confirming their knowledge and attitudes: more than 90% checked the expiration dates of probiotics and stored them in a dark, dry and cool places, and more than half kept them until the expiration date, after which they return the probiotic to the pharmacy. However, a significant percentage of respondents (42.1%) threw unused probiotics into municipal waste. Research has shown that the practice of disposing unused medicines from households differs depending on the level of economic development of the country, but that in most countries the most common practice is burning, or throwing in communal waste and in the sewer [22]. This practice is also prevalent in Serbia [23]. Considering the role of the pharmacist in pharmacotherapy and the position of being in direct contact with the patient when dispensing/selling drugs, it is an ideal opportunity and task to inform patients about the adequate disposal of unused medicines in accordance with current scientific knowledge and legal regulations [24].

### 3.2. Market Analysis

Probiotics are part of a wider category of digestive products, which is the fourth-largest over-the-counter (OTC) category as measured by IQVIA Consumer Health Report [16], with sales of USD 18 billion globally. This category is expected to outperform the OTC market as a whole, with a forcast compound annual growth rate (CAGR) of 7% for the period 2022–2025.

Health claims are a powerful tool that can often have a decisive influence on a consumer’s final choice. Since no probiotic health claims have received positive assessments by the European Food and Safety Agency (EFSA), additional active ingredients are often added to enable a wider range of authorized health claims (i.e., contributes to maintaining the normal function of the digestive and immune system). Additionally, ‘on-hold’ health claims permitted for herbal/botanical ingredients are also used for the same reason. The most persuasive producers and distributors include medicinal or therapeutic claims that are not permitted for food supplements in any form of marketing, including electronic commerce.

Since internet marketing in Serbia is not under strict governmental control, a great majority of marketed probiotics communicate via strong medicinal claims, implying efficacy and health benefits for many diseases and medical conditions in adults or children. While some of these claims are substantiated, many are based on unreliable research. The growing market for probiotics in Serbia clearly shows that physicians, pharmacists, and consumers believe that consumption of these products will provide health benefits. Almost one-quarter of all probiotics from the Serbian internet market are intended for children, with more than 10 percent being for newborns, implying an increase in routine prophylactic administration of this type of product for infants and newborns [25]. Interestingly, our results show that probiotics intended for the pediatric population were priced higher. It is important to note that, according to the national regulations in Serbia [26], requirements for health safety pertinent to the production of probiotics involve HACCP principles (Hazard Analysis Critical Control Point), together with the implementation of good hygiene and good manufacturing practices (GMP). The appropriate tests are carried out with reference to the established microbiological criteria, with no specified AMR testing requirements.

Although there is no consensus in the literature about the exact number of viable cells necessary to ensure probiotic effects, literature data show that a probiotic should provide oral doses higher than 1 × 10^9^ colony-forming units (CFUs) per day, which are required to restore and maintain the balance of bacteria, depending on the age and status of the individual [27]. The number of probiotic microorganisms in products on the Serbian market varied from 1 × 10⁶ to 4.5 × 10^11^ CFUs, and some of the products did not even declare the number of viable microorganisms.

Since members of the genus *Lactobacillus* are most widely used in all probiotic products, we tested several isolates from the best-selling probiotics in Serbia with respect to their antibiotic sensitivity. Antibiotic resistance in our study was demonstrated in the *Lactobacillus* strains on several investigated antibiotics (Table 1). Furthermore, *L. rhamnosus, L. rhamnosus* GG and *L. plantarum*, taken together, are found in almost 60% of the products included in the market analysis (Figure 4). These data seem to be of relevance and may suggest the possibility of probiotic products influencing the spread of antibiotic resistance in the population of Serbia.

### 3.3. Antibiotic Resistance

Observed on an annual basis, it is estimated that the number of deaths worldwide due to AMR is 700,000–1 million, with predictions that it will reach as many as 10 million by 2050 [27]. There is growing evidence of gene exchange between pathogenic strains and beneficial commensal bacteria in the GIT. In this way, beneficial commensal bacteria become “reservoirs” of AMR [28].

Horizontal transfer of resistance genes can occur through three mechanisms: transformation, in which foreign genetic material is obtained from the extracellular environment [29]; through the transduction mechanism, in which parts of the bacterial DNA are incorporated into the bacteriophage during replication, which subsequently infects another bacterial cell causing the transfer of genetic material [30]; or through the process of conjugation, in which cell-to-cell contact induces DNA transfer [29].

Most *Lactobacillus* species are intrinsically (naturally) resistant to aminoglycosides (gentamicin, kanamycin, streptomycin, and neomycin), ciprofloxacin, and trimethoprim, and they are susceptible to penicillin and β-lactams, chloramphenicol, tetracycline, erythromycin, linezolid, and quinupristin-dalfopristin [31,32].

It is well known that many antimicrobial agents have a strong impact on the equilibrium of lactobacilli in the human body during antibiotics therapy. As commonly advised, probiotics should be taken along with antibiotics due to the fact that antimicrobial agents also influence the microbes present in the normal flora, including lactobacilli. Metronidazole, for example, is the most commonly used antibiotic for the treatment of bacterial vaginosis. *Lactobacillus* isolates have been shown to be able to grow in the presence of metronidazole. These results suggest that selected strains could be used for a restoration therapy together with the antimicrobial bacterial vaginosis treatment. Simoes et al. [33] also studied the effect of metronidazole on the growth of vaginal lactobacilli. These authors observed partial and complete inhibition at concentration above 1000 μg/mL, while they reported a stimulating effect at concentrations between 128 μg/mL and 256 μg/mL, as well as the ability to grow in the presence of metronidazole at concentrations lower than 512 µg/mL, while lactobacilli were sensitive to most antibiotics used for treatment of urinary tract infection (UTI) (e.g., Amoxicillin + Clavulanic acid, Chloramphenicol, Nitrofurantoin, Trimethoprim-Sulfamethoxazole), and were resistant to Ciprofloxacin and Nalidixic acid, information which is of assistance in the selection of probiotics to be taken by patients during antimicrobial therapy.

Companies rarely display antibiotic sensitivity on the declaration, so data for individual strains is obtained by reviewing published papers. However, the declaration often states only the name of the species, and not the specific strain, which would be necessary for performing a literature search. This piece of information may be fundamental where AMR is concerned. For example, it is known that a specific strain—*Lymosilactobacillus reuteri* DSM 17938—was obtained through the removal of antibiotic-resistance-gene-carrying plasmids from *Lactobacillus reuteri* ATCC 55730 [34]. Additionally, no data on acquired antimicrobial resistance are available, e.g., for *L. rhamnosus* Rosell-11 [35].

The results of our experimental work and antibiotic sensitivity screening of lactobacilli isolated from the most commonly used commercial probiotics on the Serbian market (A: *Limosilactobacillus reuteri*; B: *Lactobacillus paracasei*; C: *Lactobacillus rhamnosus*; D: *Lactobacillus rhamnosus*; F: *Lactobacillus plantarum*) showed resistance to penicillin, cefoxitin, oxacillin, aztreonam and vancomycin. *Enterococcus faecium* isolated from pharmabiotics also showed resistance to the same antibiotics, except for vancomycin.

The resistance of lactobacilli to vancomycin is intrinsic and is chromosomally linked, so there is little risk of horizontal transfer of resistance genes to other genera and species of bacteria [36]. The risk of transfer is considered very low for intrinsic resistance, as well as for acquired resistance due to chromosomal mutation.

Given that lactobacilli are naturally mostly sensitive to penicillin and beta-lactam antibiotics, the detected resistance to penicillin is most likely acquired, and if the resistance genes are located on mobile genetic elements (plasmids) and transposons, in which case there is the risk implied by the possibility of horizontal transfer and the spread of resistance genes, even to conditionally pathogenic bacteria in the human gastrointestinal tract. It should be noted that the risk of resistance gene transfer is small even if the acquired resistance is the result of a gene mutation on the bacterial chromosome. Genetic tests are needed to accurately determine the resistance genes and their location.

Furthermore, according to Álvarez-Cisneros and Ponce-Alquicira [37], lactobacilli possess natural resistance to vancomycin, bacitracin, cefoxitin, metronidazole, nitrofurantoin and quinolones (ciprofloxacin, norfloxacin, nalidixic acid), and are usually resistant to trimethoprim [29]; therefore, the detected resistance of tested probiotics from the Serbian market to vancomycin, cefoxitin, metronidazole, nitrofurantoin should not carry acquired resistance genes related to mobile genetic elements, and in our opinion should therefore not represent a risk.

The determination of the genome sequences of a large number of *Lactobacillus*-type strains [38] allows the safety assessment of the genus *Lactobacillus* by surveillance of the presence of AMR genes, as well as their potential for transfer to other microorganisms. Within the limitations imposed by database quality and annotation, whole-genome sequencing (WGS) could potentially allow the identification of all possible genetic determinants of antimicrobial resistance in a microbial genome [39]. WGS could change food safety assessments, leading to a shift from phenotype-based AMR tests to genotype-based tests [40].

Our results suggest that antibiotic resistance is present in various types of lactobacilli in probiotic products, which in the case of acquired resistance can pose a safety hazard for food with probiotic strains and dietary supplements, as well as pharmacobiotics. The location of the resistance genes—on the bacterial chromosome of the tested lactobacilli or on mobile genetic elements—should be determined in future research. It seems important to include the AMR testing as a part of safety assessment for all probiotic products before they enter the market, in order to ensure beneficial effects only on the health of the human population and diminish transmission in the environment. Alternatively, our attention should be directed towards postbiotics (i.e., the use of nonviable cells or cell fragments), which have been proven to have numerous advantages without the risk of transmission of AMR [41].

## 4. Materials and Methods

### 4.1. Knowledge, Attitudes and Practice Survey

A cross-sectional observational study was conducted using a questionnaire created for the purposes of this research. The research was conducted in the period January–March 2023. Participation in the study was anonymous and voluntary. As part of the questionnaire, the respondents were provided with an informed consent form (clearly stating the goals of the study, the respondents’ right to anonymity, the use of the obtained results for scientific purposes only). The survey was conducted online, using Google Forms, and access link to the survey was distributed randomly. The research was approved by the Ethics Committee of the Faculty of Pharmacy in Novi Sad and the College of Vocational Studies in Subotica, Serbia.

### 4.2. Sample

A total of 541 respondents took part in this research: pharmacists and students of medical sciences (pharmacy, medical biochemistry, health care, nutrition and dietetics), and students of sports and the pedagogical sciences.

### 4.3. Research Instrument

The instrument used in this research was an online questionnaire. During the design period of the study, no previously validated questionnaire was found for assessing the knowledge, attitudes and practices of healthcare workers and students related to probiotics in Serbia. Therefore, the authors compiled questions based on the literature on probiotics and questionnaires from other similar studies [18,19,20]. The questionnaire was tested on a group of 10 respondents, after which the order of several questions was changed. The final questionnaire consisted of 20 questions divided into three groups: the first group of questions addressed the socio-demographic characteristics of respondents (age, gender, education and place of permanent residence), the second group of questions explored the knowledge and sources of information of the respondents, and the third group of questions investigated the attitudes and behavior of respondents in relation to the use of probiotics and professional practice.

### 4.4. Data Collection, Processing and Analysis

The questionnaire was distributed to respondents via email and social media platforms. The data that were collected through Google Forms were then exported to the Google Sheets application.

### 4.5. Market Analysis

To assess the Serbian probiotics market, we collected data on available probiotic brands on the on-line pharmacies in Serbia. From the pool of 147 registered probiotics in the Republic of Serbia, we analyzed a subsample of 113 probiotics available on the Internet market that had available information on the type and number of probiotic cultures, additional ingredients such as minerals, vitamins, prebiotics, herbal ingredients, etc. The obtained data were used to determine the declared intended use for specific age groups, the presence of health claims, the declared CFU count, the microbiological composition, and the price of the package.

### 4.6. Antibiotic Resistance Screening

To perform AMR screening, we obtained, by random purchase, the six most commonly used commercial probiotics on the Serbian market.

### 4.7. Bacterial Isolation and Identification from Probiotics

The initial suspension was prepared in such a way as to obtain as even a distribution as possible of the microorganisms found in the test sample. The initial suspension was prepared in accordance with the relevant part of ISO 6887-1. At least 10 g or 10 mL was measured into a sterile container or a sterile plastic bag, with a measurement uncertainty of ±5%, to produce a representative sample for testing. An amount of diluent equal to 9× (m/V or V/V) was added. This quantity may be measured as a mass, preferably with a measurement uncertainty of ±5%, or as a volume, with a measurement uncertainty of ±5%. The time from the end of the preparation of the initial suspension to the moment of inoculation into the culture medium did not exceed 45 min. Inoculation of the initial suspension was carried out with the loop of culture obtained over the surface of the selective agar medium (Sheep blood agar, Schaedler agar), in duplicate, under anaerobic conditions for 48 h, using AnaeroGen 2.5 L Thermo scientific (Oxoid LTD, Basingstoke, UK) in such a way as to obtain isolated smooth, round, convex, colorless colonies. Only pure cultures were used for biochemical confirmation. Further identification of the suspected bacterial colonies was carried out using matrix-assisted laser desorption/ionization–time-of-flight (MALDI–TOF) mass spectrometry (Brucker, Billerica, MA, USA).

### 4.8. Susceptibility to Antibiotics

Susceptibility to antimicrobial agents was tested using the standard disk-diffusion method on Schaedler agar (HiMedia, Thane, India) under anaerobic conditions using antibiotic discs BioRad (Marnes-la-Coquette, France), and the following antimicrobial drugs were tested: Penicilin (1 µg), Ampicilin (10 µg), Cefotaxime (30 µg), Ceftazidime (30 µg), Cefuroxime (30 µg), Cefuroxime (30 µg), Cefoxitin (30 µg), Amoxicillin + Clav. acid (20 + 10 µg), Piperacillin + Tazobactam (30 + 6 µg), Imipenem (10 µg), Meropenem (10 µg), Gentamicin (10 µg), Tobramycin (10 µg), Amikacin (30 µg), Ciprofloxacin (5 µg), Ofloxacin (5 µg), Levofloxacin (5 µg), Co-trimoxazole (25 µg), Erythromycin (15 µg), Clindamycin (2 µg), Tetracycline (30 µg), Tigecycline (15 µg), Vancomycin (30 µg), Teicoplanin (30 µg), Fusidic acid (10 µg), Nitrofurantoin (300 µg), Oxacillin (1 µg), Aztreonam (30 µg), and Chloramphenicol (10 µg), in line with the recommendation of Sharma et al. [42]. The performance for *Enterococcus* spp. only was based on the recommendations of the European Committee on Antimicrobial Susceptibility Testing (EUCAST) 2023 [43].

## 5. Conclusions

Our survey indicated several important points related to respondents’ knowledge and attitudes on probiotics. Although the vast majority of respondents wanted to learn more about probiotics, more than 40% of the participants threw unused probiotics into municipal waste. In addition, nearly half of them stated that probiotics had no side effects, while only 6.3% believed that the use of probiotics could lead to resistance to antibiotics. These data clearly suggest the need for action regarding intensified educational efforts on the possible emerging link between probiotic use and the spread of antibiotic resistance.

The probiotic market in Serbia is one of the fastest growing dietary supplement markets in southeastern Europe, delivering supplements to all population groups. Products on the market have highly variable CFU counts, their declared health claims cover numerous beneficial health effects, and they are sometimes even registered as medicines. Lactobacilli are frequently present in probiotic supplements, sold in both pharmacies and online.

The results of the experimental work showed that resistance to antibiotics is present in different types of lactobacilli in probiotic products. This can potentially represent a safety hazard for food with probiotic strains, dietary supplements, as well as medicines. The risk of using probiotics, regardless of their beneficial health effects, should be taken into account in the future. In order to diminish the spread of AMR genes in the population and the environment, updated regulations for probiotics that include AMR testing should be established, and guidelines for their proper use and disposal should be put into place.

## Figures and Tables

**Table 1:** Results of antibiotic susceptibility testing of isolates from commercial products on the Serbian market. (d—diameter [mm]; int—interpretation).

Antibiotic	A * Limosilactobacillus *reuteri* 12/28 **	B * Lactobacillus *paracasei* 14/28 **	C * Lactobacillus *rhamnosus* 16/28 **	D * Lactobacillus *rhamnosus* 15/28 **	E * Enterococcus *faecium* 15/28 **	F * Lactobacillus *plantarum* 11/28 **
	d/int	d/int	d/int	d/int	d/int	d/int
Penicilin	6/R	6/R	6/R	6/R	6/R	6/R
Ampicillin	26/S	35/S	24/S	32/S	6/R	34/S
Cefotaxime	6/R	6/R	6/R	6/R	6/R	32/S
Ceftazidime	25/S	6/R	6/R	6/R	6/R	24/S
Cefuroxime	32/S	6/R	6/R	25/S	6/R	35/S
Cefoxitin	6/R	6/R	6/R	6/R	6/R	6/R
Amoxicillin + Clav. acid	25/S	40/S	40/S	36/S	6/R	37/S
Piperacillin + Tazobactam	6/R	32/S	35/S	32/S	6/R	25/S
Imipenem	30/S	27/S	6/R	32/S	6/R	50/S
Meropenem	30/S	6/R	6/R	6/R	6/R	40/S
Gentamicin	40/S	20/S	18/I	25/S	17/I	25/S
Tobramycin	26/S	14/R	10/R	18/I	6/R	18/I
Amikacin	35/S	23/S	20/S	30/S	20/S	22/S
Ciprofloxacin	22/S	32/S	18/I	6/R	31/S	6/R
Ofloxacin	15/I	24/S	17/I	6/R	28/S	6/R
Levofloxacin	18/I	30/S	17/I	6/R	32/S	10/R
Co-trimoxazole	6/R	6/R	6/R	6/R	6/R	37/S
Erythromycin	45/S	36/S	40/S	30/S	15/I	42/S
Clindamycin	50/S	36/S	25/S	20/S	6/R	23/S
Tetracycline	6/R	30/S	6/R	23/S	35/S	16/I
Tigecycline	15/I	20/S	16/I	27/S	30/S	25/S
Vancomycin	6/R	6/R	6/R	6/R	24/S	6/R
Teicoplanin	6/R	6/R	6/R	6/R	23/S	6/R
Fusidic acid	6//R	6/R	6/R	6/R	20/S	6/R
Nitrofurantoin	6/R	6/R	6/R	6/R	25/S	6/R
Oxsacillin	6/R	6/R	6/R	6/R	6/R	6/R
Aztreonam	6/R	6/R	6/R	6/R	6/R	6/R
Chloramphenicol	36/S	27/S	25/S	32/S	35/S	32/S

* A, B, C, D, F—probiotics registered as nutritional supplements; E—pharmacobiotic. d—radius of bacterial growth (mm). R—resistant; S—sensitive; I—intermediate. **—Ratio of resistant/overall tested. Shaded cells in the table indicate resistance to the tested antibiotic.

## Data Availability

No new data were created or analyzed in this study. Data sharing is not applicable to this article.

## Supplementary Materials

The following supporting information can be downloaded at: https://www.mdpi.com/article/10.3390/antibiotics12081281/s1, answers to all survey questions represented as Figures S1 to S7.
