# Supplementary material for: Antibiotic Resistance and Probiotics: Knowledge Gaps, Market Overview and Preliminary Screening
Source: Antibiotics (Basel). 2023 Aug 3;12(8):1281. doi: 10.3390/antibiotics12081281 (PMC10451169; doi:10.3390/antibiotics12081281)
Supplement: Supplementary file 1 [file antibiotics-12-01281-s001.zip › antibiotics-2504903-supplementary.pdf]

SUPPLEMENTARY MATERIALS. Survey results.

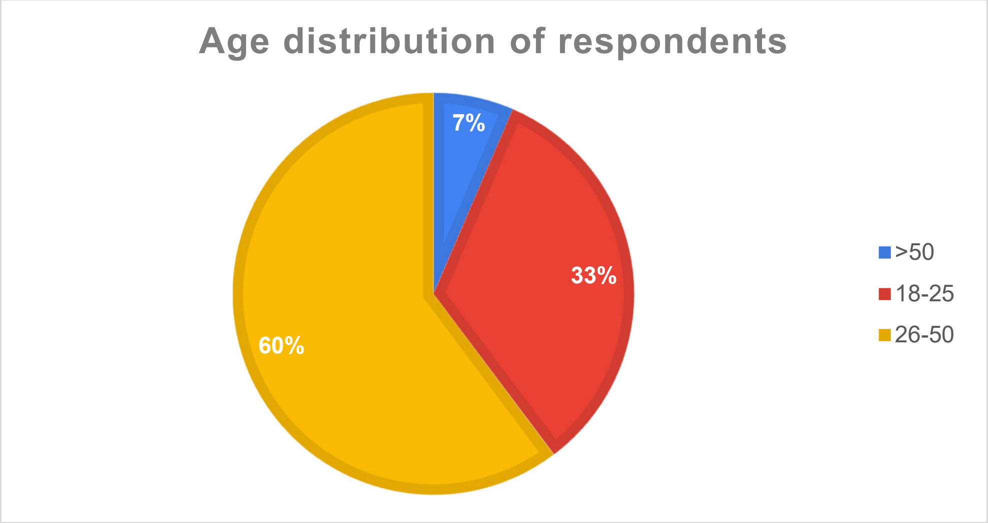

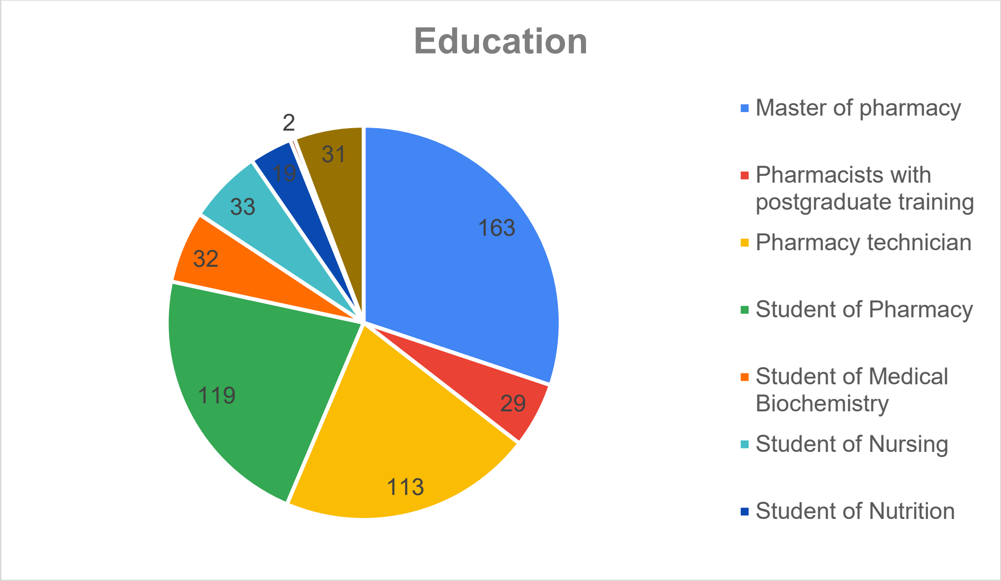

Figure S1 Age distribution and education of the 541 respondents in the survey.

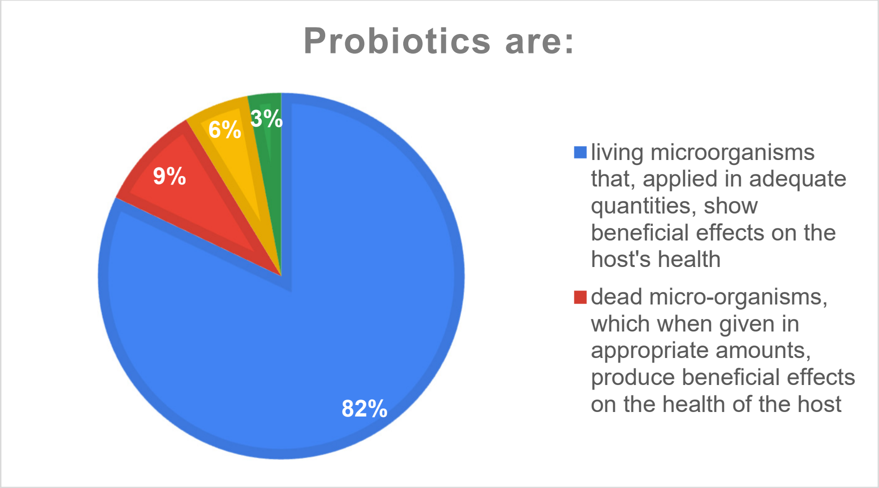

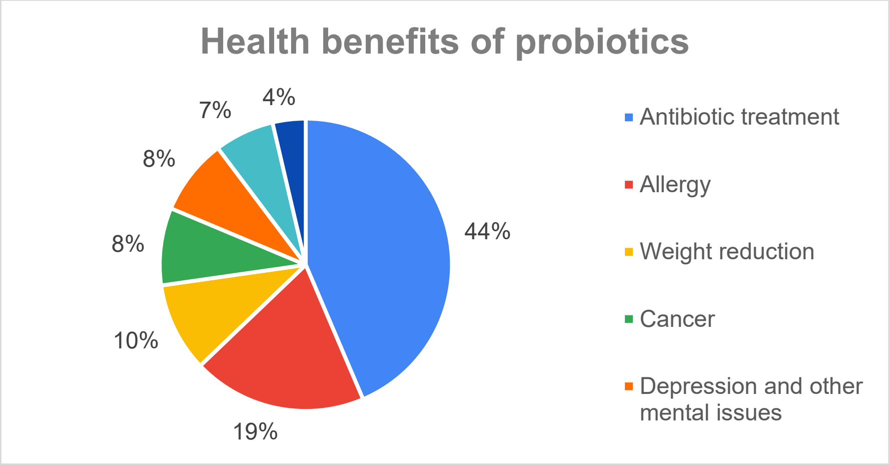

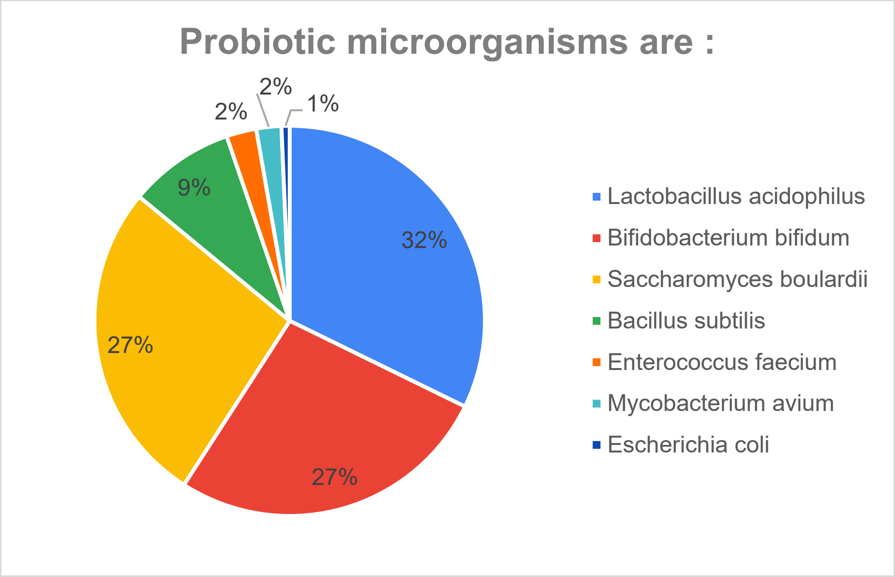

Figure S2. Basic knowledge on probiotic microorganisms and the health benefits.

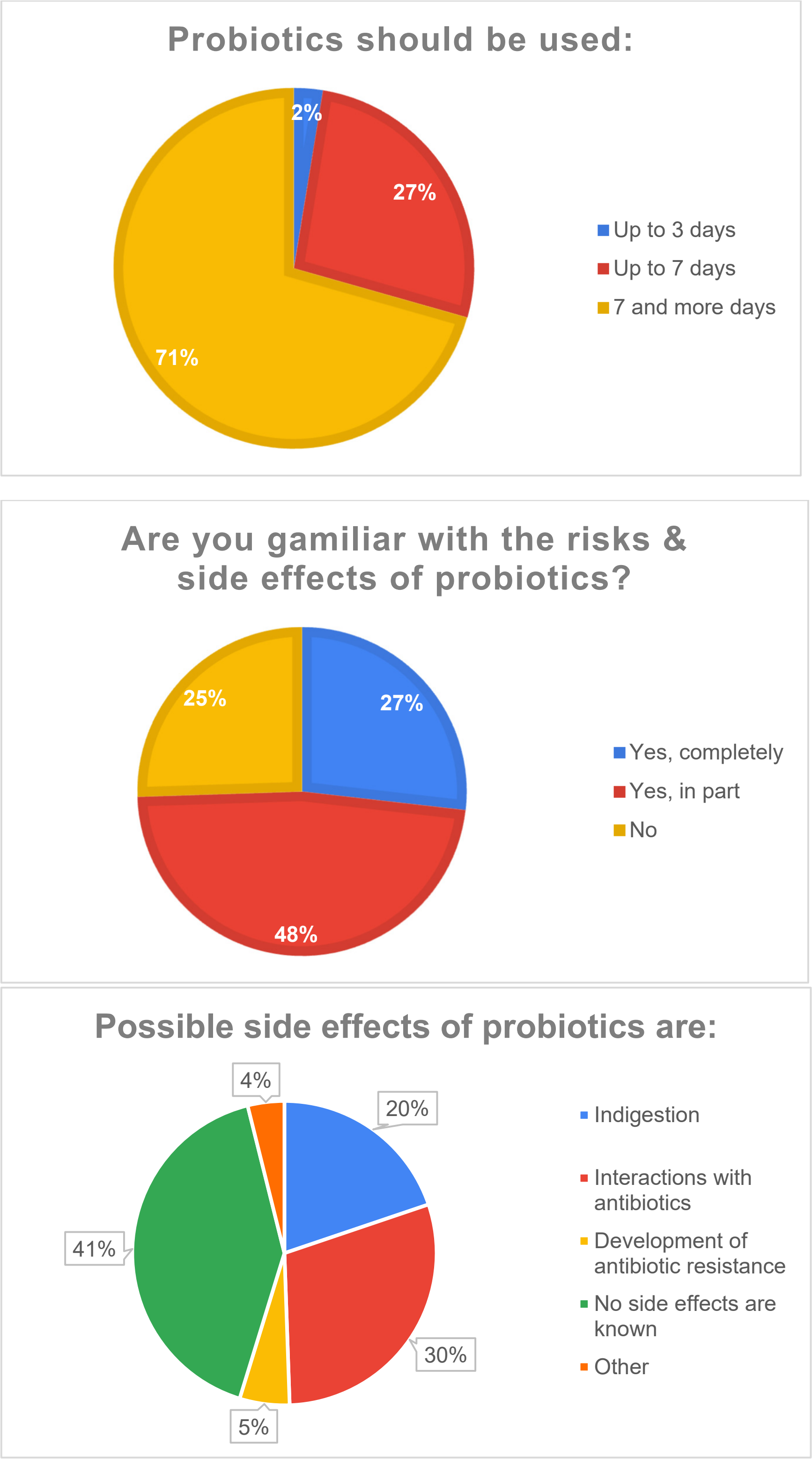

Figure S3. Use and side effects of probiotics.

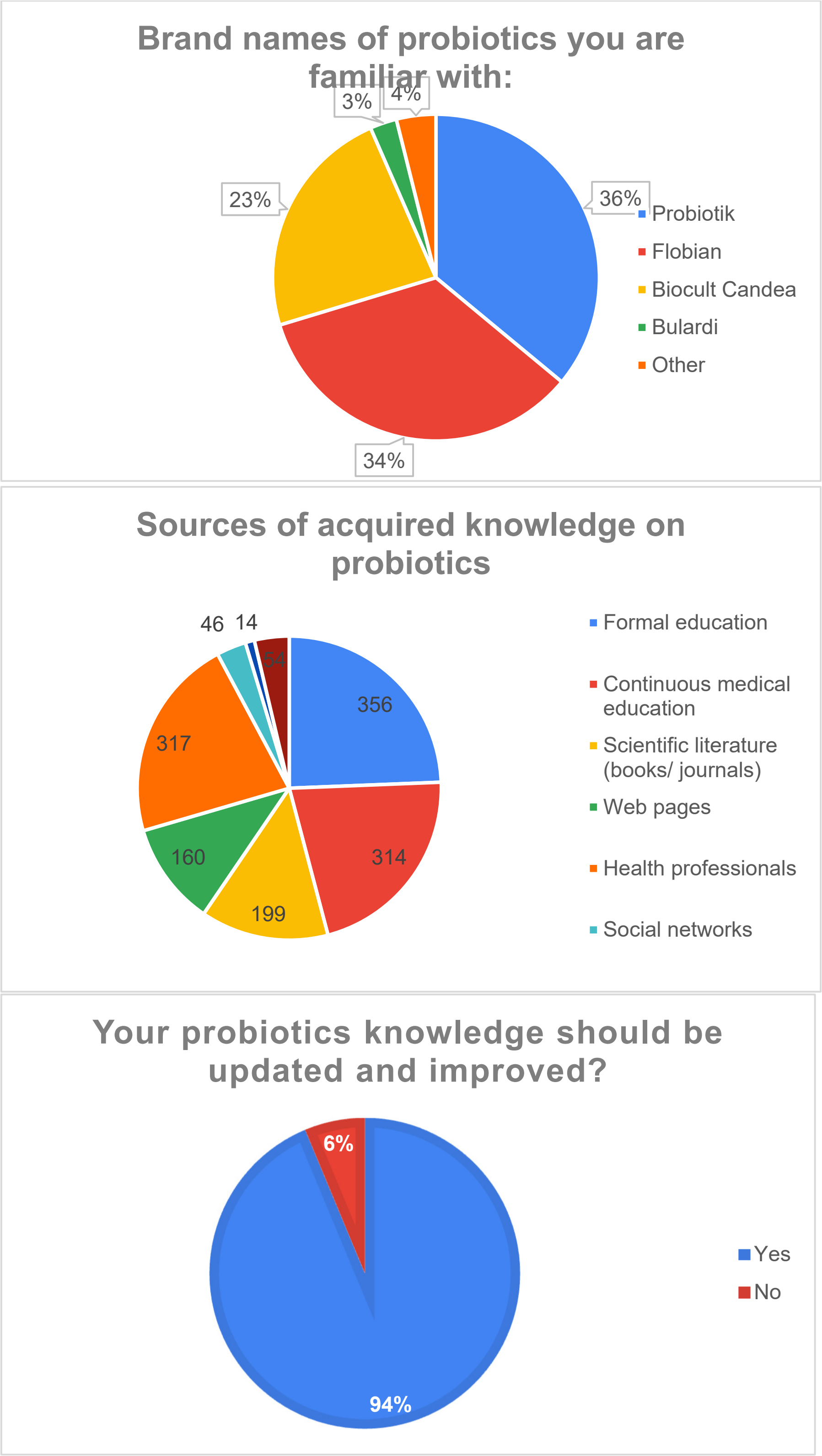

Figure S4. Familiar brand names and knowledge sources & updates.

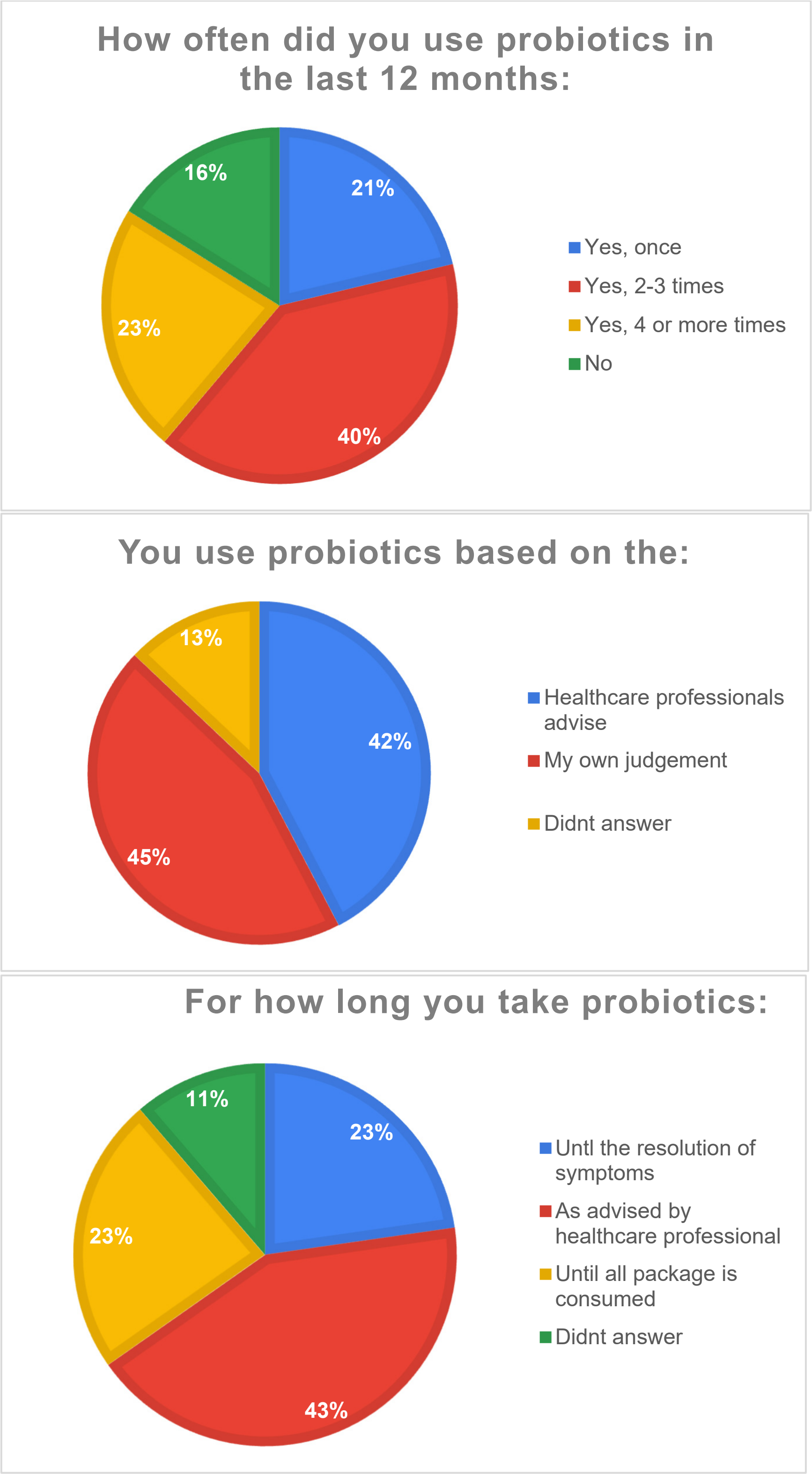

Figure S5. Use of probiotics

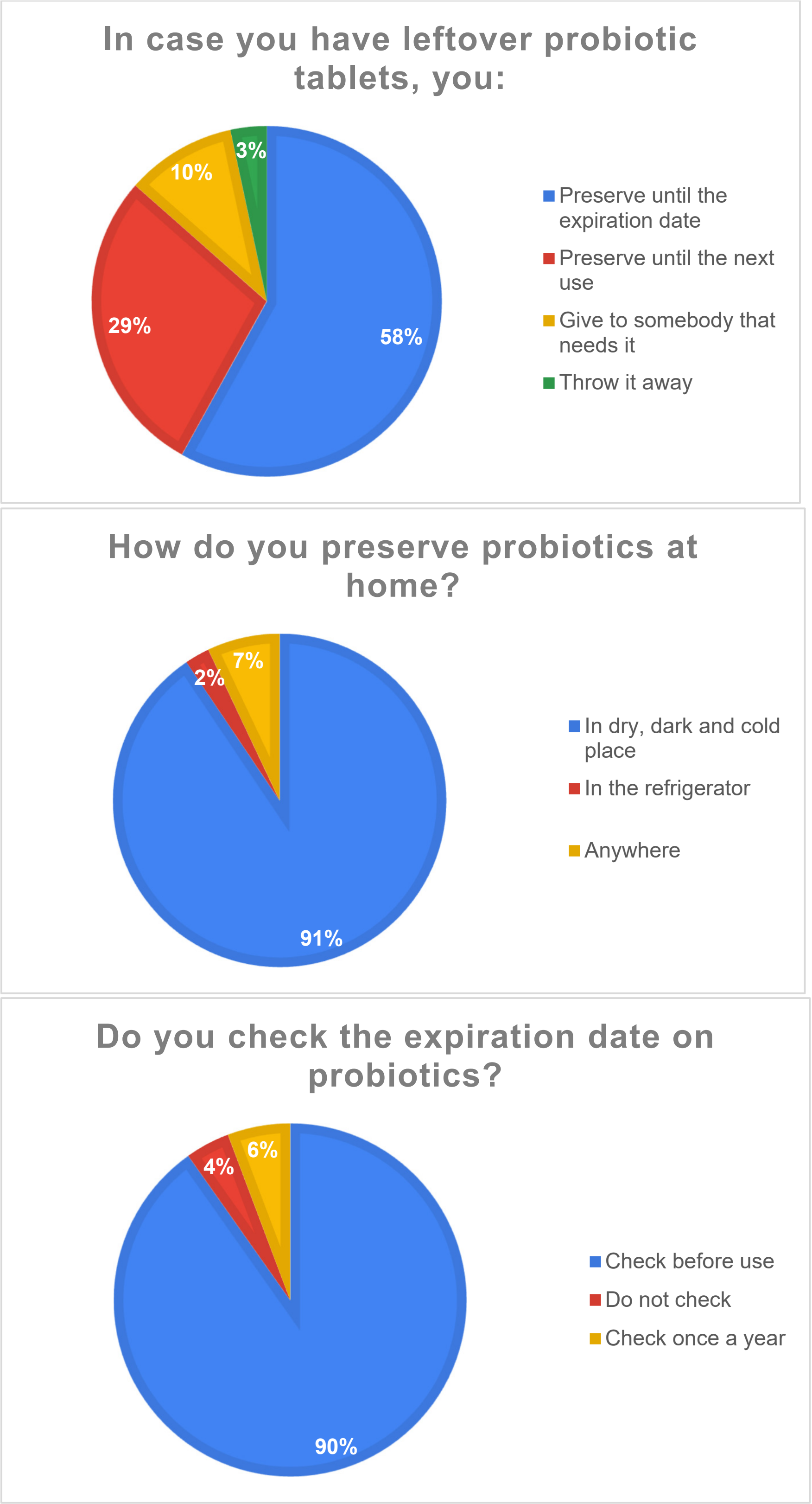

Figure S6. Preservation of probiotics.

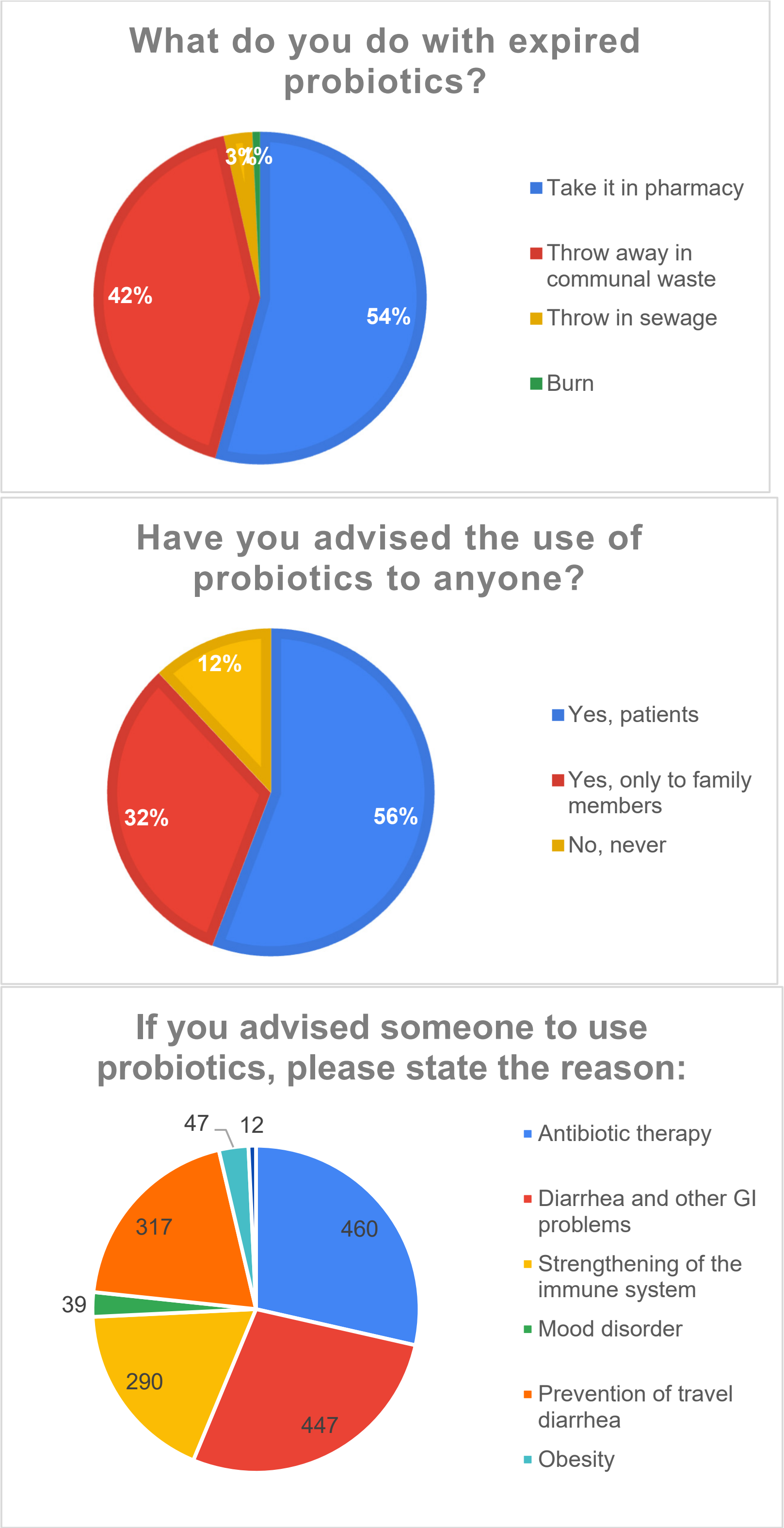

Figure S7. Disposal and giving advice on probiotics use.
